# Optimization of Injection Molding Process for High-Strength and Lightweight Back Rest of Firefighters Using Carbon Fiber Composites of Long Fiber Thermoplastic with Flame Retardants

**DOI:** 10.3390/ma18051112

**Published:** 2025-02-28

**Authors:** Kyoung-Jae Min, Joon-Hyuk Song, Hyun Tak, Bhum-Keun Song

**Affiliations:** Korea Carbon Industry Promotion Agency, 110-11 Banryong-ro, Jeonju 54853, Republic of Korea; bluemy@kcarbon.or.kr (K.-J.M.); songjh@kcarbon.or.kr (J.-H.S.); htak@kcarbon.or.kr (H.T.)

**Keywords:** carbon fiber LFT composite, oxygen respirators back rest, weight reduction, Taguchi method, flame-retardant

## Abstract

This study focuses on reducing the weight of oxygen respirators in firefighters’ personal protective equipment (PPE), which currently accounts for about 56% of the total weight. The heavy PPE, weighing between 20 and 25 kg, restricts movement and can lead to musculoskeletal injuries. To address this, the study investigates using a carbon fiber-reinforced composite for the backrest of the oxygen respirator to reduce weight while maintaining strength. The backrest was fabricated using a long-fiber thermoplastic (LFT) composite made with PA66 resin and 30 wt.% carbon fiber content. Initially, the injection-molding process conditions were identified to achieve a tensile strength of 85 MPa or higher. Additionally, flame retardants were added to improve fire resistance, with AF-480 at 5 wt.% found to be the best option. Subsequently, optimal injection conditions were set by fabricating the back rest with the composite by applying the Taguchi method to satisfy the required tensile strength. As a result, the composite material achieved a 12.8% weight reduction while maintaining the required strength. This development is expected to significantly improve firefighter safety, leading to more effective firefighting and reduced human and property damage.

## 1. Introduction

**Figure 1:**
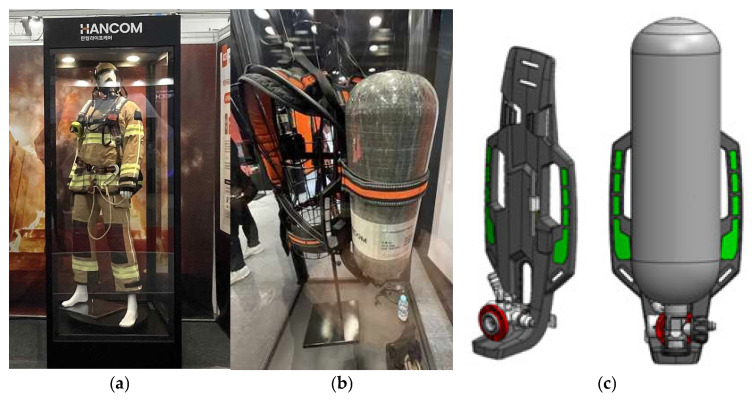
Personal protective equipment (PPE for firefighters); (**a**) firefighter mannequin wearing a PPE (**b**) respirator and back rest set (**c**) design model of respirator and back rest set.

Firefighters dispatched to fire sites for rapid extinguishment typically carry heavy personal protective equipment (PPE) weighing between 20 and 25 kg, which includes oxygen respirators and specialized gear, as shown in Figure 1. Firefighters wear heavyweight PPE, as shown in Figure 1a. The development of technology for high-strength and lightweight PPE for firefighters operating at the forefront of fire sites is essential for ensuring their safety and enhancing their ability to respond effectively to fires. Oxygen respirator is a critical component that accounts for about 56% of the weight of firefighters’ PPE, and thus, it is a prime target for weight reduction. Among the components of the PPE, shown in Figure 1b, the back rest is crucial and is currently manufactured from conventional engineering plastics through the injection molding process. Due to its low tensile strength, the thickness of the back rest must be increased to support the heavy weight of PPE, which increases the weight of the back rest. To endure the heavy weight of PPE, the tensile strength and tensile modulus of the back rest must be maintained up to the existing ones. Figure 1c is a design model using carbon fiber composite to reduce the weight of the back rest.

The utilization of fiber-reinforced polymer (FRP) has experienced rapid growth in recent years and is expected to rise further. This trend is attributed to the advantages that FRP provides, including high performance in structural applications, particularly in the fields of automotive, aerospace, maritime, civil infrastructure, and wind energy [1,2,3,4]. FRP composites have gradually replaced metal and ceramic matrix composites in some areas due to low density, high specific stiffness and specific strength values, high fatigue durability, high corrosion resistance, excellent insulation, and low thermal expansion [5,6,7].

Composites generally exhibit poor fire performance due to low impact damage tolerance, anisotropic properties, and the unique flammability of the polymer matrix. Consequently, these characteristics have constrained the application of composites despite the high demand in the field of firefighting [8,9,10].

Injection molding facilitates the production of detailed three-dimensional plastic components by injecting various polymer materials into cold molds at high pressure. This technology is widely utilized for solid polymer processing across various industries. Various injection foaming processes derived from conventional forming technology have been employed for foam production. Nevertheless, foam injection molding is a complex process that encounters significant challenges, thereby limiting potential applications during the development phase. To address these issues and produce components with specific functions, numerous specialized injection molding processes have been developed over the past several decades. Alternative processing technologies, such as Microcellular [11,12], Profoam [13,14], Stages Moulding [15,16], and Pull and Foam [17,18], have been introduced.

The implementation of these technologies enables a substantial reduction in the weight of various components; however, significant modifications in process control or mold design are necessary to achieve components that meet the desired requirements. Therefore, the industrial application of these technologies often fails to ensure the best performance of the produced components. An effective method for manufacturing components that meet established requirements involves traditional foam injection molding, which can be optimized by adjusting process parameters such as materials, additives, and processing conditions [19,20].

This study focused on producing a back rest via injection molding using carbon fiber (CF) long fiber thermoplastic (LFT) to enhance strength through the application of CFs. The objective of weight reduction necessitated a transition from engineering plastics to a CF-reinforced composite aimed at improving tensile strength.

Oxygen respirators account for approximately 56% of the total weight of firefighters’ PPE, thereby rendering weight reduction of this equipment critical. Among the components of the respirator, the back rest represents a key element necessitating weight reduction; it is constructed from conventional engineering plastics through an injection molding process. Due to its low strength, the thickness of the back rest is increased to support the load of the oxygen tank and adjacent equipment, contributing to its overall weight. This study investigates methods to reduce the weight of the back rest while preserving its required properties by substituting conventional engineering plastics with a carbon fiber (CF)-reinforced composite. Thus, the back rest was fabricated using a composite that applied the long-fiber thermoplastic (LFT) of PA66 resin with a CF content of 30% through the injection process. Additionally, flame retardants were incorporated to maintain performance in fire scenarios, thereby enhancing the capability of firefighters to respond effectively in the field.

First, a composite incorporating LFT of PA66 resin with a CF content of 30 wt.% was utilized, and injection conditions were selected to achieve a tensile strength of 85 MPa or greater and a tensile elastic modulus of 10 GPa or higher. Tensile test specimens were prepared by incorporating 1 wt.%, 3 wt.%, and 5 wt.% of three types of flame retardants, each containing inorganic compounds of phosphorus and nitrogen that pose no harm to human health. Subsequently, a flame retardant exhibiting high flame retardancy was identified through flame retardant testing.

Second, an experiment was performed to determine the optimal injection conditions of the back rest for firefighters who applied the LFT by applying the Taguchi method.

Taguchi’s design of experiments is effective for efficient experimental design and cost reduction. That is, since it is inefficient to try all possible variable combinations in experimental design, Taguchi’s technique reduces the number of experiments by using the orthogonal array method while systematically analyzing the variables of complex processes, thereby obtaining effective results.

In particular, we confirmed the conditions required for producing the back rest with optimal performance, a tensile strength of 85 MPa or higher, and a tensile elastic modulus of 10 GPa or greater.

## 2. Materials and Methods

### 2.1. Materials and Preparation of Flame Retardants

The carbon fiber (CF) composite masterbatch utilized in this study was LFT of Xiamen LFT Composite Plastic Co., Ltd. (Xiamen, China), which is composed of 30 wt.% CF, with a CF length ranging from 10 to 12 mm, a density of 1.8 g/cm^3^, and a tensile strength between 2500 and 2600 MPa. Polyamide 66 (PA66) resin of 1.14 g/cm^3^ density and 80–90 MPa tensile strength was employed due to its high chemical resistance, wear resistance, and ability to maintain strength and stiffness at elevated temperatures. PA66 composites containing 30 wt.% CF maintain the material’s viscosity at an appropriate level, allowing smooth mold-filling during injection molding. These viscosity characteristics provide high mechanical strength and stiffness without compromising processability [21].

The selected flame retardants were non-halogenated compounds, as halogens are detrimental to human health. The candidates included ESCON-360AC, ESCON-775, and AF-480. The information on the characteristics of each flame retardant is listed as follows in Table 1.

The thermal decomposition temperatures for each flame retardant are as follows: 295 °C for ESCON-360AC, 280 °C for ESCON-775, and 280 °C for AF-480. ESCON-360AC exhibits superior flame-retardant performance, attributed to its highest content of phosphorus and nitrogen, as well as its largest particle size. In particular, ESCON-775 demonstrates adequate flowability, facilitated by its high phosphorus content and relatively smaller particle size, whereas AF-480 is noted for its high dispersibility during injection owing to its small particle size.

The flame retardants were mixed with the PA66 resin-CF composite masterbatch using an X-axis rotary mixer. The mixing time of each flame retardant was set to 60 min at 30 RPM to ensure sufficient mixing. The addition of flame retardants diminishes the flowability of the resin intended for injection. This addition typically occurs within the range of 1–5 wt.%, as exceeding a specific threshold can impede the injection process due to reduced flowability. In this study, each flame retardant was incorporated at concentrations of 1 wt.%, 3 wt.%, and 5 wt.% of the total resin weight, respectively. The LFT composite was applied for the weight reduction of the back rest using conventional PA66, and each of AF-480, ESCON-360AC, and ESCON-775 was added by 1 wt.%, 3 wt.%, and 5 wt.%, respectively, for flame retardancy. The specimens for the flame resistance test were prepared through the injection molding process of the LFT composite.

### 2.2. Injection-Molding Process and Conditions

The specimens were fabricated using an injection mold designed to produce tensile specimens corresponding to each content of the master batch prepared in Section 2.1. The specimens were produced in compliance with the ASTM D 3039 standard. The injection conditions for specimen preparation are detailed, as shown in Table 2.

The cylinder temperatures were set to 300 °C based on a maximum section of 300 °C by referring to the melting temperature range of 277 to 299 °C. The injection speed was set within the range of 75–95 mm/s. The Injection pressures were set at from 70 to 120 MPa based on injection pressure specifications of 69–124 MPa provided by the raw material manufacturer. The maximum nozzle temperature is 300 °C.

### 2.3. Flame Resistance Test

To determine the optimal flame retardant and its appropriate concentration for application to the back rest, a flame resistance test was conducted on each specimen. The flame resistance tests on the back rest were conducted in accordance with clause 6.11.2.2 of EN-137, the flame engulfment test standard for respirators, as there is no direct standard of flame resistance test for back rests.

The testing apparatus consisted of a propane storage tank equipped with control instrumentation and a precise pressure gauge, a flame backflow prevention device, six propane burners with adjustable height, and a mechanism enabling the metal standard iron model to move vertically and horizontally. Each propane control valve of the six burners was fully opened, while the air control valves of each burner were closed. The pressure gauge of the propane output device was calibrated to ensure that the total flow rate of propane supplied to the six burners was (21 ± 0.5) L/min. Prior to measuring the flame temperature, the burners were accurately positioned, and the burner flame was adjusted to limit the test temperature to 950 ± 50 °C. The test specimens were subsequently placed on the holder and exposed to the flame for 10 s. Following the test, the after-flame period was measured while the specimens remained in position.

### 2.4. Optimization of Injection-Molding Process Conditions for the Back Rest Using the Taguchi Method

The weight of the back rest, which serves as PPE for firefighters, was reduced by altering the material from PA66 to CFRTP incorporating LFT. The injection process was employed for the CFRTP back rest utilizing LFT.

As such, various injection molding conditions can influence the quality of the product during the injection molding process. The quality of the product is contingent upon multiple controllable factors during the operation of the injection molding machine, including injection speed, injection pressure, injection temperature, cooling time, injection time, mold temperature, cooling temperature, and back pressure.

During injection molding, temperature control encompasses the cylinder temperature necessary for melting resin, the temperature of the mold, and the cooling temperature and duration. The thermal characteristics of the resin significantly influence the appearance, quality, and physical properties of the final product. The molten resin exhibits fluidity, which is contingent upon viscosity, a property that varies with the temperature of the molten resin. A lower viscosity enhances fluidity, facilitating the filling of the cavity within the mold and improving internal deformation and appearance. Consequently, the temperature of the molten resin is elevated. However, if the resin is exposed to excessive heat or temperatures exceeding its thermal deformation threshold, it may undergo carbonization or thermal decomposition. Therefore, meticulous temperature management is essential. Moreover, when the mold temperature is elevated, the resin can be charged rapidly. Nevertheless, thermal deformation may occur post-filling, necessitating an increase in cooling time. The mold temperature influences the surface condition of the product; hence, it is maintained as high as feasible while accounting for the risk of thermal decomposition. Prolonged exposure to elevated temperatures may result in defects, such as flash, carbonization, and sink marks. If the temperature is low or lasts over a short period of time, short shots, weld lines, and gloss differences may occur.

The injection pressure refers to the pressure per unit area exerted on the molten resin at the screw end of the injection molding machine. The injection pressure, denoted as P (MPa), is the product of the cross-sectional area of the cylinder and the hydraulic pressure, P0 (MPa). To determine the cross-sectional area of the cylinder, A (cm^2^), the diameter of the cylinder, D (cm), is analyzed, as expressed in Equation (1). The injection pressure can be calculated by multiplying the derived cross-sectional area of the cylinder by the hydraulic pressure, as illustrated in Equation (2). Notably, the injection pressure is reduced by 30% to 50% through the fluidic device in the mold, a factor that should be considered [22].

(1) $$\frac{\pi }{4}\times D^{2}=A\;$$

(2) $$A\times P_{0}=P$$

A high injection speed is preferable, as it is advantageous for maintaining the constant density and strength of the product by ensuring the uniform temperature of the molten resin. Given that the injection pressure and speed may vary based on the geometry or cross-sectional area of the fluidic device in the mold, it is essential to design the fluidic device with these considerations during the product and mold design phase. The injection time is established according to the charging and appearance quality of the product and can be primarily divided into filling time and packing time. If the packing time is shorter than the gate sealing time, the contraction cannot be sufficiently compensated due to the backflow of the molten resin in the cavity through the unsealed gate. This condition may lead to sink marks or short shots. Conversely, if the packing time is excessively long, production efficiency decreases as a consequence of the increased overall injection molding cycle time. This phenomenon is influenced by the size and geometry of the gate, as well as the injection temperature and pressure [23].

Cooling characteristics vary based on the flow rate of the coolant, the cooling time, and the type of cooling employed. Notably, the cooling time represents the longest duration within the injection molding cycle. Preparation for cooling occurs prior to injection, with coolant continuously flowing until its supply is discontinued. Following injection and subsequent sealing of the gate, the resin formed inside the cavity contracts during the cooling phase. The contraction of the molten resin is affected by the cooling rate, coolant temperature, and flow rate. Furthermore, the cooling time is determined by several factors, including the cylinder temperature of the injection molding machine, the mold temperature, the thermal dissipation of the resin, and the size and geometry of both the fluidic device and the injection-molded product [23].

**Figure 2:**
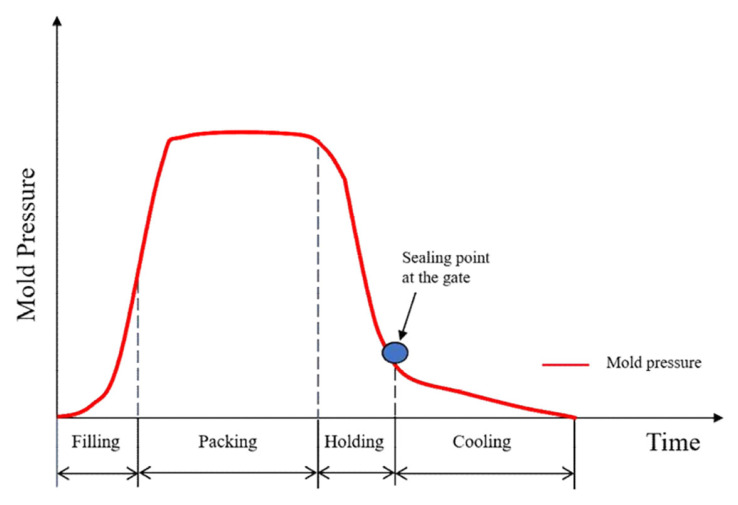
Mold cycle time and relationship between pressure. Source: A Study on the Injection Molding Machine and Injection Condition for Improving the Quality of Plastic Products [23].

Figure 2 depicts the pressure inside the mold during the injection molding process time. The pressure inside the mold is highest during packing, and this packing pressure is maintained. Following the sealing point at the gate, as illustrated in the figure, the resin within the cavity exhibits no backflow, and the cooling process is executed [23].

Considering the factors associated with these injection conditions, suitable injection parameters for achieving a tensile strength of 85 MPa or higher, which is necessary for the back rest, were selected. Based on the identified injection molding conditions, the back rest was fabricated through the injection molding process under the condition of Table 3.

### 2.5. Taguchi’s Array and Injection Molding Process

In general, CF LFT composites have the effect of increasing tensile strength and reducing weight [23]. However, when flame retardants are included in this composite, this demonstrates a reduction in tensile strength and alterations in injection characteristics. In this study, the Taguchi method was employed to identify the optimal injection-molding process conditions for CF LFT composites containing flame retardants to obtain the required tensile strength.

The Taguchi method, developed by Dr. Genichi Taguchi, a Japanese statistician and engineer, serves as a technique for optimizing technology. In 1980, Dr. Taguchi achieved significant success by spearheading the development of production technology for semiconductors at the Bell Research Institute in the United States. Since that time, the Taguchi method has gained worldwide recognition and is referred to as the Taguchi Method Robust Design. In Japan, it is termed Quality Engineering.

Taguchi’s orthogonal design constitutes a design of experiments (DOE) approach that is particularly effective for minimizing time and cost. The Taguchi method advocates for the application of the signal-to-noise (S/N) ratio to engineering design problems to ascertain quality characteristics [24].

The S/N ratio has three characteristics. The principle of “smaller is better,” “nominal is best”, and “larger is better” is articulated by Taguchi (1990) in “Introduction to Quality Engineering” (New York: McGraw-Hill) [24].

In this study, the S/N ratio for tensile strength was calculated under the premise that a higher S/N ratio is preferable for determining the effects of process parameters on tensile strength values and for identifying the optimal point.

The S/N ratio is calculated using Equation (3) [24].

(3) $$S/N=-10\cdot \log\left[\frac{1}{n}\left(\sum_{i=1}^{n}\frac{1}{y_{i}^{2}}\right)\right]$$

where *i* represents the measurement and *n* denotes the number of samples for each test.

In a three-level orthogonal array, each column possesses two degrees of freedom. Given that interaction effects occur in two columns, and one column has two degrees of freedom, the interaction effects collectively account for four degrees of freedom.

$$L_{3^{n}}\left(3^{\left(3^{n}-1\right)/2}\right)$$

3: number of factors.

$3^{n}$: total number of experiments (N) (number of rows).

$3^{\left(3^{n}-1\right)/2}$: number of factors that can be arranged (number of columns).

The three-level Taguchi orthogonal array is denoted as L_9_ (3^4^).

In total, nine experiments were conducted, with four columns, each possessing two degrees of freedom. The accompanying table designates the values 0, 1, and 2 to signify the levels of each factor.

The optimal injection conditions for tensile strength and tensile modulus selection by Taguchi DOE are as follows.

The injection process parameters determined using the Taguchi method for tensile strength optimization are listed in Table 4. The cylinder temperatures were set to 260, 280, and 300 °C at 20 °C intervals based on a maximum section of 300 °C by referring to the melting temperature range of 277 to 299 °C, which was the injection temperature condition provided by the manufacturer of the raw material. The injection speed was set within the range of 75–95 mm/s. The pressures were set at 70, 95, and 120 MPa at 25 MPa intervals, based on a maximum value of 120 MPa. This range was determined by referencing the injection pressure specifications of 69–124 MPa provided by the raw material manufacturer. The nozzle temperature varied from 260–300 °C. The injection process was performed using JINHWA GLOTECH (Cheonan, Republic of Korea)’s STL-800 (800t class).

### 2.6. Specimen Preparation

The specimens of tensile strength testing were prepared by machining the samples into dimensions of ASTM D 3039 (2.8 mm × 15 mm × 250 mm). Table 5 is an orthogonal array, $L_{9}\left(3^{4}\right)$, of the Taguchi orthogonal array, and Table 6 contains the process parameters according to the Taguchi orthogonal array $L_{9}\left(3^{4}\right)$. Zwick/Roell’s universal testing machine served as the testing equipment, conducting tests via displacement control at a rate of 2 mm/min. Average values were obtained from seven points of data, excluding the maximum/minimum from nine experiments.

## 3. Results

### 3.1. The Flame Retardant Test Results

**Figure 3:**
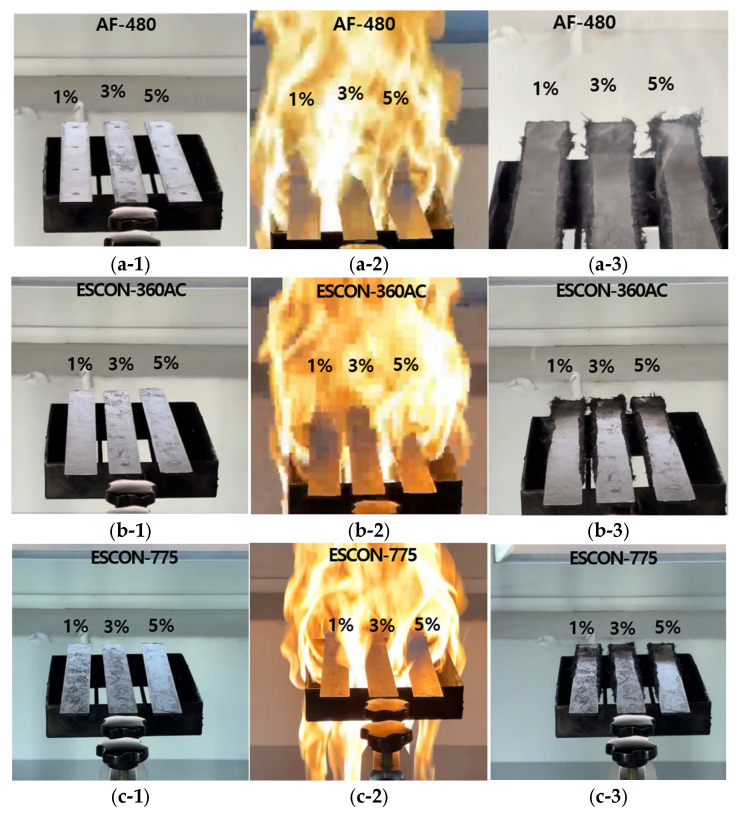
Flame resistance test images by flame retardant: (**a-1**) AF-480 specimens before the test; (**a-2**) AF-480 specimens during the test; (**a-3**) AF-480 specimens after the test; (**b-1**) ESCON-360AC specimens before the test; (**b-2**) ESCON-360AC specimens during the test; (**b-3**) ESCON-360AC specimens after the test; (**c-1**) ESCON-775 specimens before the test; (**c-2**) ESCON-775 specimens during the test; (**c-3**) ESCON-775 specimens after the test.

The flame-retardant test results and images are presented in Table 7 and Figure 3.

From Table 7, the results indicated that AF-480 exhibited the shortest time of residual flame. Therefore, the flame resistance test was additionally conducted on specimens containing 5 wt.% of each flame retardant for final verification. The subsequent test results are shown in Table 8.

Flame retardants can have the disadvantage of reducing flowability during injection molding. That is, there are cases where the results from the flame resistance test are irregular. The conditions for uniform flame resistance test results were selected. In the case of AF-480, the flame-retardant performance tended to increase as the flame-retardant content increased. However, Escon-360 and Excon-775 did not show any consistent regularity. The time of residual flame of ESCON-360AC 5 wt.% was 8 s 79 in Table 7 and 4 s 61 in Table 8. The time of residual flame of ESCON-775 5 wt.% was 1 s 43 in Table 7 and 5 s 28 in Table 8. Since the deviation of the results was large even under the same conditions, these were not selected as a flame retardant in this paper.

**Figure 4:**
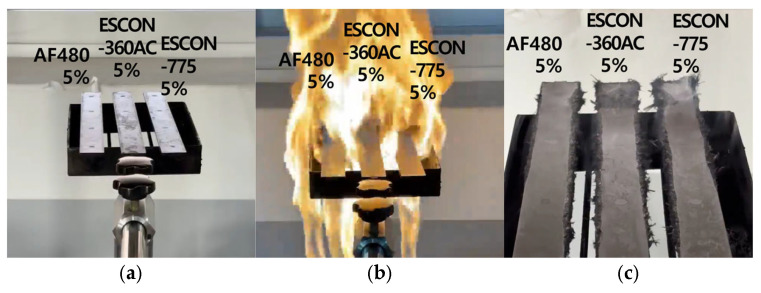
Flame resistance test images by flame retardant (5 wt.%): (**a**) specimens before the test; (**b**) specimens during the test; (**c**) specimens after the test.

The test results finally confirmed that AF-480 had the shortest time of residual flame. From the Table 8 and Figure 4, the addition of AF-480 5 wt.% to the LFT of PA66/CF30 wt.% was selected and applied for testing under back rest injection-molding process conditions. The results of the flame resistance test indicated that AF-480 at a 5 wt.% concentration exhibited the highest flame resistance. Consequently, the back rest was fabricated through injection molding utilizing the LFT containing 5 wt.% AF-480 as a flame retardant.

### 3.2. The Back Rest Fabricated Through Injection Molding

**Figure 5:**
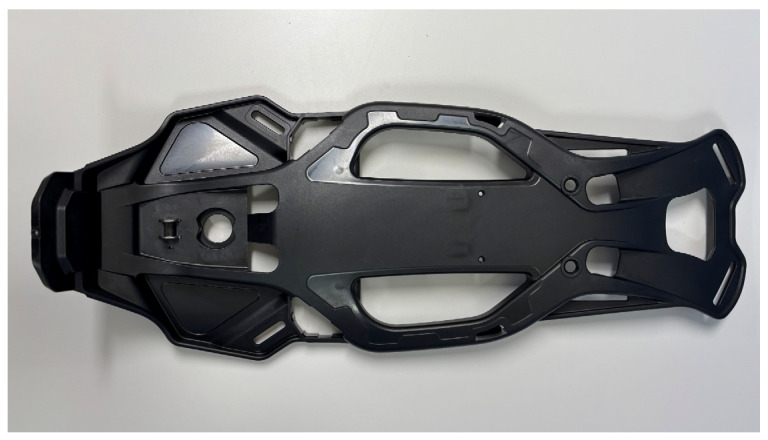
The back rest fabricated through injection molding.

The back rest fabricated through injection molding is illustrated in Figure 5. The back rest constructed from conventional engineering plastics weighed 780 g, whereas the back rest utilizing the developed LFT composite weighed 680 g.

Table 9 presents the results of tensile strength and Tensile elastic modulus under the specified experimental conditions.

### 3.3. Tensile Strength Test Results

**Figure 6:**
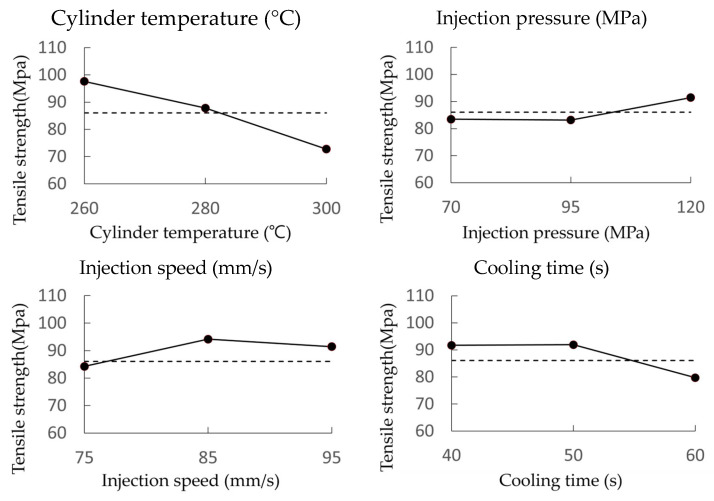
Effects of each parameter on tensile strength.

The test results indicate that tensile strength is inversely proportional to both cylinder temperature and cooling time while being directly proportional to injection pressure. The effects of each parameter on tensile strength are detailed in Figure 6 and Table 10.

These results are corroborated by the average values presented in Table 10 (the underlined values represent the optimal combination of parameters that maximize tensile strength: A0, B2, C1, and D1). At DOE level 0, the maximum tensile strength is observed at the optimal cylinder temperature. The data presented in the graph and table confirm that cylinder temperature has the most significant impact on tensile strength.

### 3.4. Tensile Elastic Modulus Test Results

**Figure 7:**
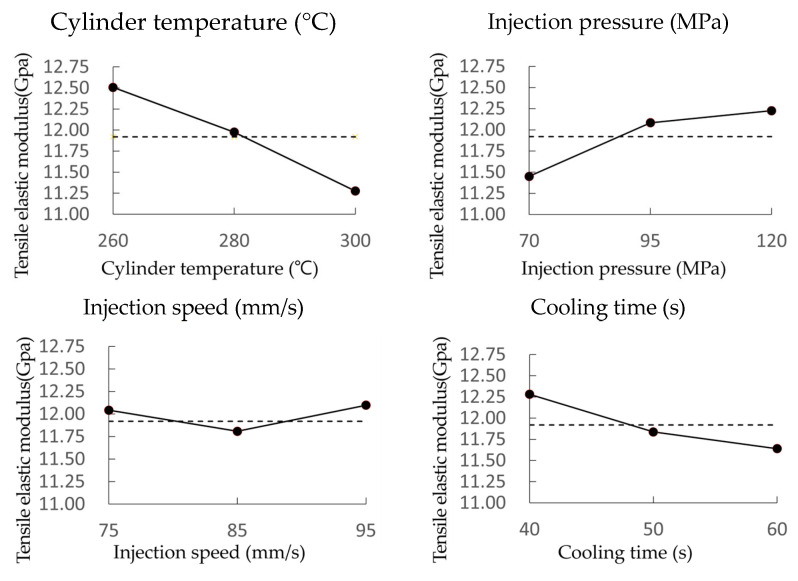
Effects of each parameter on the tensile elastic modulus.

The test results demonstrate that the tensile elastic modulus is inversely proportional to both cylinder temperature and cooling time while being directly proportional to injection pressure. The effects of each parameter on the tensile elastic modulus are outlined in Figure 7 and Table 11.

These results are supported by the average values in the table, where the underlined values represent the optimal combination of parameters that maximize the tensile elastic modulus: A0, B2, C2, and D0. Furthermore, at DOE level 0, the maximum tensile elastic modulus is observed at the optimal cylinder temperature. The graph and table data illustrate that the tensile elastic modulus is predominantly influenced by cylinder temperature, with secondary effects noted from injection pressure and cooling time.

## 4. Conclusions

This research focused on the optimization of the injection molding to reduce the weight of the back rest, a component of personal equipment for firefighters. The Taguchi method was used to optimize the parameters of the injection molding process. With this method, it achieved weight reduction while maintaining the required tensile strength and tensile modulus despite the addition of flame retardants. In order to reduce the weight of the back rest of PPE, the injection molding process was performed using LFT of PA66 resin with a CF content of 30 wt.% substitutes the conventional engineering plastics. And, through a flame retardance test, an appropriate flame retardant was selected as 5 wt.% AF-480.

The optimal injection molding process conditions for the LFT composite were determined using the Taguchi orthogonal array method. The cylinder temperature emerged as the parameter exerting a significant influence on both the tensile strength and tensile elastic modulus. The optimal injection conditions for achieving maximum tensile strength and tensile elastic modulus were identified as a cylinder temperature of 260 °C, an injection pressure of 70 MPa, an injection speed of 75 mm/s, and a cooling time of 40 s. Under these conditions, the tensile strength reached 98.9 MPa, exceeding the required tensile strength, while the tensile elastic modulus measured 12.51 GPa.

The back rest for firefighters was subsequently fabricated using these injection conditions. Thus, a weight reduction of 12.8% was achieved.

This research aims to secure lightweight and flame retardancy while satisfying tensile strength and tensile modulus by applying carbon fiber composites. The development of high-strength and lightweight PPE for firefighters at disaster sites will significantly contribute toward effective fire extinguishing activities, which will ensure the safety of firefighters by reducing human casualties and property damage. In the future study, impact and fatigue properties will be researched.

## Figures and Tables

**Table 1:** The characteristics of flame retardants.

Category	AF-480	ESCON-360AC	ESCON-775
Appearance	White powder	Gray powder	White powder
Phosphorus content	Min 18% (*w*/*w*)	Min 20% (*w*/*w*)	Min 22% (*w*/*w*)
Nitrogen content	Min 10% (*w*/*w*)	Min 14% (*w*/*w*)	Min 10% (*w*/*w*)
	10 μm (average, D50)	20 (average, D50)	15 μm (average, D50)
Thermal decomposition	Min 350 °C (2% weight loss)	Min 295 °C (2% weight loss)	280 °C
Density	Approx. 1.45 (g/mL, at 25 °C)	Approx. 1.90 (g/mL, at 25 °C)	1.75 (g/mL, at 25 °C)
Water content	Max 0.20% (*w*/*w*)	Max 0.50% (*w*/*w*)	Max 0.10% (*w*/*w*)

**Table 2:** The conditions of injection-molding process for flame resistance test specimens.

Conditions	Values
Cylinder temperature (Front/intermediate/rear)	0/300/300 °C
Injection speed	75–95 mm/s
Injection pressure	70–125 MPa
Nozzle temperature	300 °C

**Table 3:** The conditions of injection-molding process for the back rest with PA66/CF30 wt.%/AF-480 5 wt.%.

Factors	Value
Cylinder temperature	260/280/300 °C
Injection speed	75/85/95 mm/s
Injection pressure	70/95/120 MPa
Cooling time	40/50/60 s

**Table 4:** The injection molding process parameters and levels for Taguchi method.

Factor	Parameter	Unit	0	Level 1	2
A	Cylinder temperature	°C	260	280	300
B	Injection speed	mm/s	75	85	95
C	Injection pressure	MPa	70	95	120
D	Cooling time	s	40	50	60

**Table 5:** An orthogonal array $L_{9}\left(3^{4}\right)$ of Taguchi orthogonal array.

No.	Cylinder Temperature	Injection Pressure	Injection Speed	Cooling Time
1	0	0	0	0
2	0	1	1	1
3	0	2	2	2
4	1	0	1	2
5	1	1	2	0
6	1	2	0	1
7	2	0	2	1
8	2	1	0	2
9	2	2	1	0

**Table 6:** Process parameters according to Taguchi orthogonal array $L_{9}\left(3^{4}\right)$.

No.	Cylinder Temperature (°C)	Injection Pressure (MPa)	Injection Speed (mm/s)	Cooling Time (s)
1	260	70	75	40
2	260	95	85	50
3	260	120	60	60
4	280	70	85	60
5	280	95	95	40
6	280	120	75	50
7	300	70	95	50
8	300	95	75	60
9	300	120	85	40

**Table 7:** The results of flame resistance test for PA66/CF 30% with flame retardants ESCON-360AC, ESCON-775, and AF-480.

Category	AF-480			ESCON-360AC			ESCON-775		
	1%	3%	5%	1%	3%	5%	1%	3%	5%
Area of residual flame	Partial Edge	Full Edge	Full Edge	Partial Edge	Full Edge	Full Edge	Full Edge	Full Edge	Full Edge
Time of residual flame (second)	5.17	4.83	1.34	16.55	2.97	8.79	4.99	4.51	1.43

**Table 8:** The results of flame resistance test for PA66/CF 30 wt.% with flame retardants 5 wt.% of ESCON-360AC, ESCON-775, and AF-480, respectively.

Category	AF-480 5 wt.%	ESCON-360AC 5 wt.%	ESCON-775 5 wt.%
Area of residual flame	Full Edge	Full Edge	Full Edge
Time of residual flame (second)	1.34	4.61	5.28

**Table 9:** The results of tensile strength and tensile elastic modulus.

No.	Cylinder Temperature (°C)	Injection Pressure (MPa)	Injection Speed (mm/s)	Cooling Time (s)	Tensile Strength (MPa)	Tensile Elastic Modulus (GPa)
1	260	70	75	40	98.9	12.5
2	260	95	85	50	97.6	12.5
3	260	120	60	60	96.2	12,5
4	280	70	85	60	93.0	11.1
5	280	95	95	40	84.3	12.5
6	280	120	75	50	86.2	12.3
7	300	70	95	50	58.7	10.7
8	300	95	75	60	67.6	11.3
9	300	120	85	40	91.9	11.8

**Table 10:** Tensile strength results according to the parameters.

Factor		A	B	C	D
Level	0	97.6 MPa	83.5 MPa	84.3 MPa	91.7 MPa
	1	87.8 MPa	83.2 MPa	94.2 MPa	91.9 MPa
	2	72.7 MPa	91.5 MPa	91.5 MPa	79.7 MPa
Delta		24.8	8.3	14.4	12.2
Rank		1	4	2	3

**Table 11:** Tensile elastic modulus results according to the parameters.

Factor		A	B	C	D
Level	0	12.5 GPa	11.4 GPa	12.0 GPa	12.3 GPa
	1	12.0 GPa	12.1 GPa	11.8 GPa	11.8 GPa
	2	11.3 GPa	12.2 GPa	12.1 GPa	11.6 GPa
Delta		1.2	0.8	0.3	0.6
Rank		1	3	4	2

## Data Availability

The data presented in this study are available on request from the corresponding author due to privacy.
